# Intravital microscopy of collective invasion plasticity in breast cancer

**DOI:** 10.1242/dmm.034330

**Published:** 2018-08-23

**Authors:** Olga Ilina, Leonard Campanello, Pavlo G. Gritsenko, Manon Vullings, Chenlu Wang, Peter Bult, Wolfgang Losert, Peter Friedl

**Affiliations:** 1 Department of Cell Biology, Radboud Institute for Molecular Life Sciences, Radboud University Medical Center, PO Box 9101, 6500HB, Nijmegen, The Netherlands; 2 Department of Physics, Institute for Physical Science and Technology, University of Maryland, College Park, MD 20742, USA; 3 Department of Pathology, Radboud University Medical Center, PO Box 9101, 6500HB, Nijmegen, The Netherlands; 4 Cancer Genomic Centre, 3584CG, Utrecht, The Netherlands; 5 David H. Koch Center for Applied Research of Genitourinary Cancers, The University of Texas MD Anderson Cancer Center, Houston, TX 77030, USA

**Keywords:** Carcinoma invasion, Intravital microscopy, Actin dynamics

## Abstract

Cancer invasion programs are adaptive by switching between metastatic collective and single-cell dissemination; however, current intravital microscopy models for epithelial cancer in mice fail to reliably recreate such invasion plasticity. Using microimplantation of breast cancer spheroids into the murine mammary fat pad and live-cell monitoring, we show microenvironmental conditions and cytoskeletal adaptation during collective to single-cell transition *in vivo*. E-cadherin-expressing 4T1 and E-cadherin-negative MMT tumors both initiated collective invasion along stromal structures, reflecting invasion patterns in 3D organotypic culture and human primary ductal and lobular carcinoma. Collectively invading cells developed weakly oscillatory actin dynamics, yet provided zones for single-cell transitions with accentuated, more chaotic actin fluctuations. This identifies collective invasion *in vivo* as a dynamic niche and efficient source for single-cell release.

## Abstract (precis)

**Summary:** We developed an orthotopic breast cancer model in mice to monitor, by intravital microscopy, the transitions between collective and single cell invasion and their differential stromal interaction and actin dynamics.

## INTRODUCTION

Progression and fatal outcome of breast cancer disease result from the emergent ability of cancerous cells to invade tissue, cope with complex tissue microenvironments, and adapt their metastatic dissemination programs by switching between collective and individual-cell migration programs (Cheung and Ewald, 2016). Early steps and molecular drivers of single-cell dissemination of epithelial cancers *in vivo* were identified by intravital microscopy (IVM) in rodent models, including oncogenic mutations in Wnt, EGFR, P53 (also known as Trp53) and TGF-β signaling pathways (Fumagalli et al., 2017; Giampieri et al., 2009; Kedrin et al., 2008). IVM has further revealed how tumors co-evolve with the reactive tumor stroma and undergo anatomic, molecular and functional reprogramming, and the significance of tumor-associated macrophages directing local invasion and systemic dissemination (Friedl and Alexander, 2011; Harney et al., 2015).

In epithelial cancers assessed by histopathological analysis, collective cell patterns are abundant at the invasion front (Bronsert et al., 2014; Cheung et al., 2013; Khalil et al., 2017). Collective invasion occurs in cell groups or strands connected and coordinated by adherens and other cell-cell junctions that mediate multicellular polarity, actomyosin contractility and cell-cell signaling (Friedl and Alexander, 2011). Subsequent to local epithelial cancer invasion, persisting cell-cell interactions can support collective metastasis by tumor cell clusters circulating in peripheral blood and collective organ colonization (Aceto et al., 2014; Cheung et al., 2016). However, to date, IVM models of epithelial cancers, including breast cancer and colorectal cancer, have not been able to reliably detect and mechanistically interrogate collective invasion (Fumagalli et al., 2017; Gligorijevic et al., 2014; Kedrin et al., 2007). As a consequence, *in vivo* insights into collective invasion in epithelial cancers, its guidance by tissue structures, and the mechanisms enabling transitions between collective and single-cell invasion remain lacking.

Here, we applied microsurgical implantation of multicellular breast cancer spheroids into the mammary fat pad, followed by intravital mammary window imaging. From our model, we identified principles of collective invasion, transitions to single-cell dissemination and associated modulation of cytoskeletal states.

## RESULTS

### Implantation and window-based monitoring of growth and metastasis in mammary tumors

**Fig. 1.**
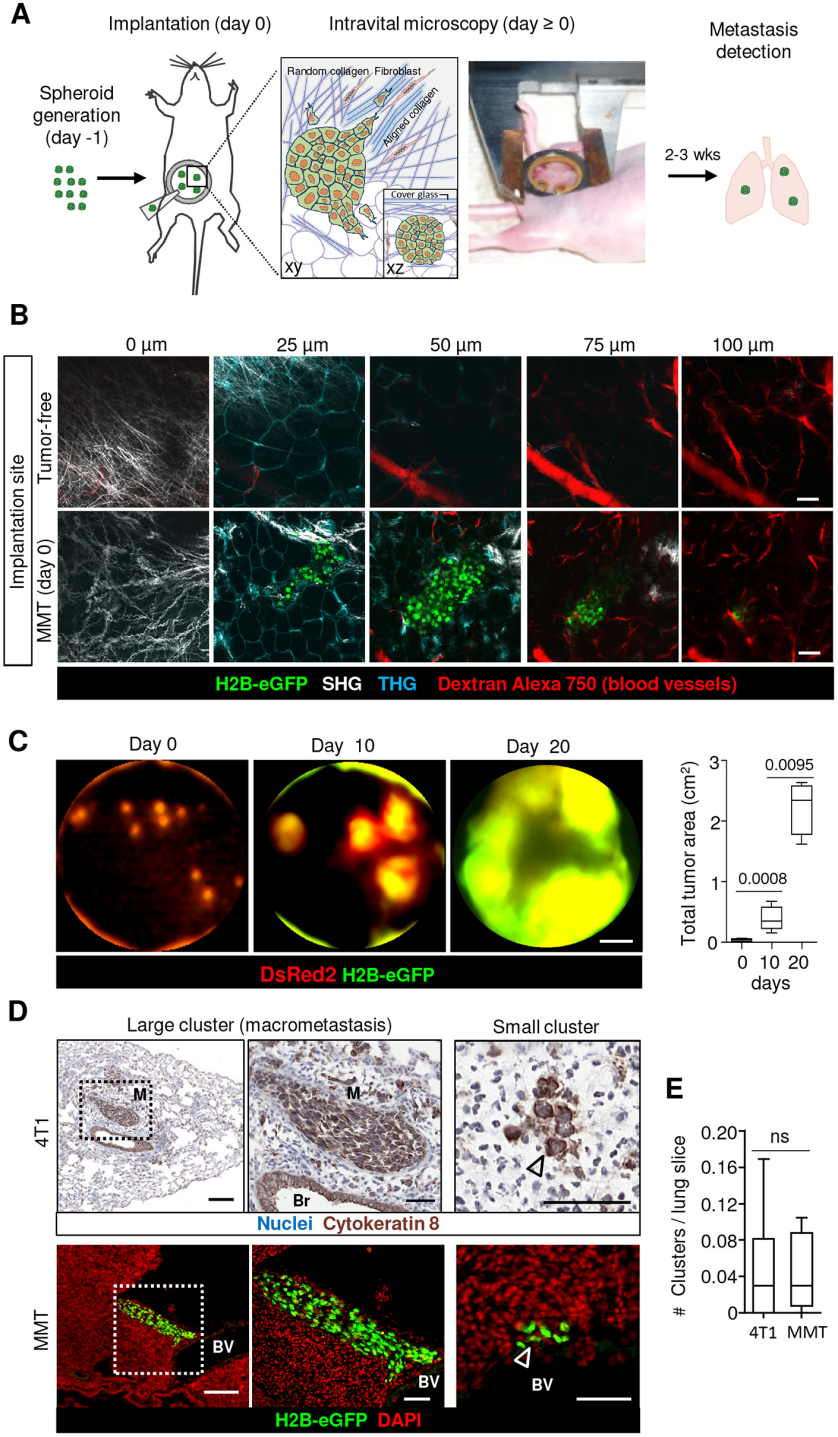
**Mammary imaging model to monitor tissue invasion and subsequent metastasis formation.** (A) Schematic representation of the experimental design with spheroid implantation into the mammary fat pad and subsequent metastasis detection. The main invasion-guiding tissue structures within the mammary fat pad are represented. An image of the mouse after surgery mounted with a custom-made holder for intravital microscopy is also shown. (B) *Z*-stack of images showing tissue structure in the mammary fat pad before spheroid implantation (upper row). *Z*-stack of images of MMT tumor spheroid after implantation into the mammary fat pad (lower row). The image depth is indicated for every image. (C) Multifocal tumor growth monitored by whole-body fluorescence imaging and quantification of multifocal MMT tumor growth. Data represent 6 mice with 7-9 tumors per mouse. Box plots display the median (black line), 25/75 percentiles (boxes) and maximum/minimum (whiskers). *P*-values were obtained by the Mann–Whitney test. (D) Macro- and micrometastasis in the lungs of mice 2-3 weeks after 4T1 (upper row) or MMT (lower row) tumor implantation in the mammary fat pad. Arrowheads indicate cluster of carcinoma cells. Br, bronchus; BV, blood vessel; M, metastasis. (E) Frequency of multicellular metastatic foci per lung slice. Minimum size of 4T1 and MMT metastatic cluster was considered as ≥3 cells. On average, 74-85 (4T1) and 85-124 (MMT) slices per lung were analyzed. Metastatic clusters were detected in 5 of 6 (MMT) and 7 of 10 (4T1) mice. Box plots display the median (black line), 25/75 percentiles (boxes) and maximum/minimum (whiskers) from 6 (MMT) and 10 (4T1) mice. *P*-values were obtained by the Mann–Whitney test. Scale bars: 50 μm (B); 2 mm (C); 200 μm (D, left column), 50 μm (D, middle and right columns).

To create a model for monitoring collective invasion of breast cancer cells by intravital microscopy, the mammary imaging model (Kedrin et al., 2008) was adapted for microimplantation of multicellular spheroids at the collagen-containing border of the 4th mammary fat pad (Fig. 1A,B). To maximize throughput, up to 10 spheroids were implanted in the same fat pad (Fig. 1C), mimicking multifocal disease (Hofmeyer et al., 2012). Implanted mouse mammary 4T1 and MMT spheroids contained intercellular junctions including E-cadherin (4T1), β-catenin and p120 catenin (4T1, MMT) (Fig. S1A-C). The integrity of spheroids, connective and adipose tissue, and vascular networks were preserved after implantation (Fig. 1B; Fig. S1D), consistent with minimally invasive microsurgery. Multifocal tumors grew exponentially for periods up to 3 weeks (Fig. 1C; Fig. S1E,F) and developed spontaneous micro- and macrometastasis to the lungs (Fig. 1D,E). In contrast to spheroids, 4T1 cells injected as suspension established bulky tumors without signs of collective invasion (Fig. S1G). Thus, the mammary imaging model recapitulates the growth of primary carcinoma lesions followed by distant metastasis.

### Intravital microscopy of collective invasion and individualization

**Fig. 2.**
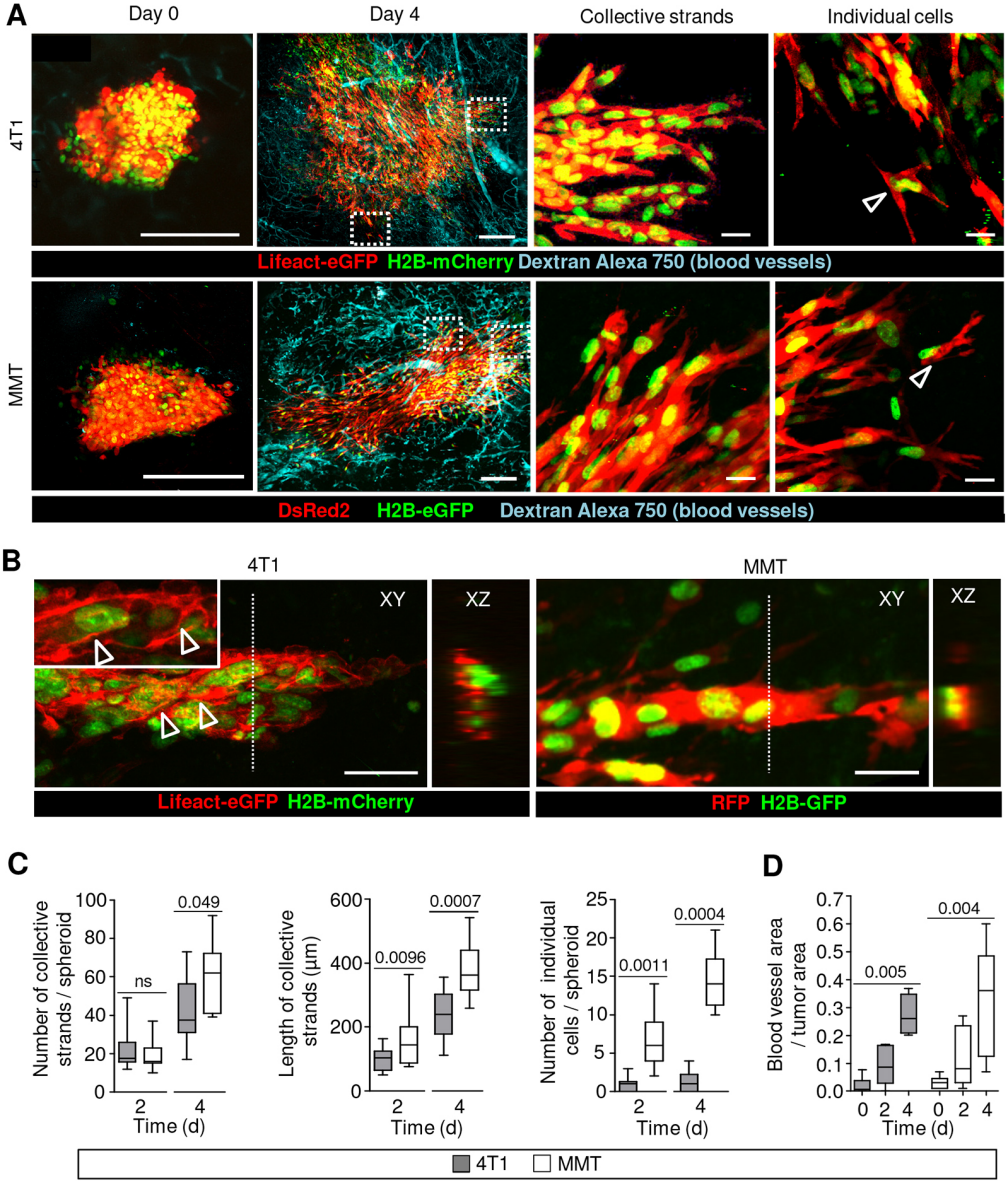
**Collective and individual-cell invasion patterns and angiogenesis in the mammary tumor imaging model.** (A) Time-course of 4T1 (upper row) and MMT (lower row) tumor growth and angiogenesis monitored by 2-photon microscopy. Overview images are montages of separate images, representing maximum intensity projections of *z*-stacks obtained with 5 µm intervals. Dotted line boxes show close-ups of collectively invading strands and single cells. Arrowheads indicate single cells. (B) Collective invasion of 4T1 (left) and MMT (right) cells in the mammary fat pad 4 days after spheroid implantation. Horizontal (XY) and orthogonal (XZ) projections of *z*-stacks. Dashed lines mark the position of cross-section planes for orthogonal views. Arrowheads indicate enrichment of Lifeact-eGFP along cell-cell junctions. (C) Number of collectively invading strands per tumor, length of collectively invading strands and number of individually invading cells per tumor. Collective strands were identified as groups of connected tumor cells (based on GFP or DsRed2 signal) radially protruding from multicellular spheroids. Cell-cell contacts were detected as nuclear H2B-eGFP signal, with either Lifeact-eGFP along cell-cell boundaries (4T1) or continuous DsRed2 signal (MMT) between nuclei in single sections from *z*-stacks. Data represent 9-16 collective strands and 9-10 single cells from 7-12 tumors from 4 mice per cell line. (D) Blood vessel network area normalized to tumor area. Data represent 4-11 tumors from 4 mice per cell line. Box plots in C and D display the median (black line), 25/75 percentiles (boxes) and maximum/minimum (whiskers). *P*-values were obtained by the Mann–Whitney test. Scale bars: 200 μm (A, overviews); 25 μm (A, areas from dotted line boxes shown magnified on the right); 50 μm (B).

Collective cell invasion in both 4T1 and MMT tumors initiated within 1-2 days after implantation, irrespective of E-cadherin expression (Fig. 2A; Fig. S1A). 4T1 cells expressing E-cadherin invaded as solid collective strands with detectable leader cells (Fig. 2A, upper row, and B, left). MMT cells which lacked E-cadherin but expressed N-cadherin (Fig. S1A,B), as reported (Firlej et al., 2008), invaded as less tightly organized, but nonetheless collective, strands or networks with multicellular organization and head-to-tail cell alignment (Fig. 2A, lower row, and B, right). Similar collective invasion was obtained when 4T1 and MMT spheroids were implanted into 3D collagen matrix culture, where cells within multicellular invasion strands retained cell-cell junctions enriched in filamentous actin and adherens junction proteins E-cadherin (4T1) and β-catenin (MMT) (Fig. S2A). The ratio of membrane/cytoplasm E-cadherin fluorescence was ∼1.5 along cell-cell junctions in collective strands and decreased to ∼0.5 in detached single cells (Fig. S2B).

Both 4T1 and MMT cells were able to detach and migrate individually with elongated, spindle-shaped morphology (Fig. 2A, right column) and downregulated E-cadherin at the cell surface (Fig. S2B). Detached single cells were closer to collective strands compared with the spheroid main mass, indicating that single-cell detachment is more likely to occur from collective invasion sites compared with the main tumor mass (Fig. S2C). Individualization was more abundant in MMT compared with 4T1 tumors, which developed more frequent tip cells and detached cells (Fig. 2C). The inverse association between cell individualization and E-cadherin expression in the spheroid implantation model *in vivo* is consistent with the observed increased single-cell release in 3D organotypic culture of MMT compared with 4T1 spheroids (Fig. S2D), and in patient samples from human lobular compared with ductal breast carcinoma (Fig. S2E) (Khalil et al., 2017).

Thus, grafted 4T1 and MMT tumors develop predominantly collective invasion of the mammary tissue, and this is consistent with the dominating collective invasion patterns found in human samples of both E-cadherin-positive ductal and E-cadherin-negative lobular breast carcinoma (Bronsert et al., 2014; Cheung et al., 2013; Khalil et al., 2017).

### Tissue-guiding structures of mammary carcinoma cells

**Fig. 3.**
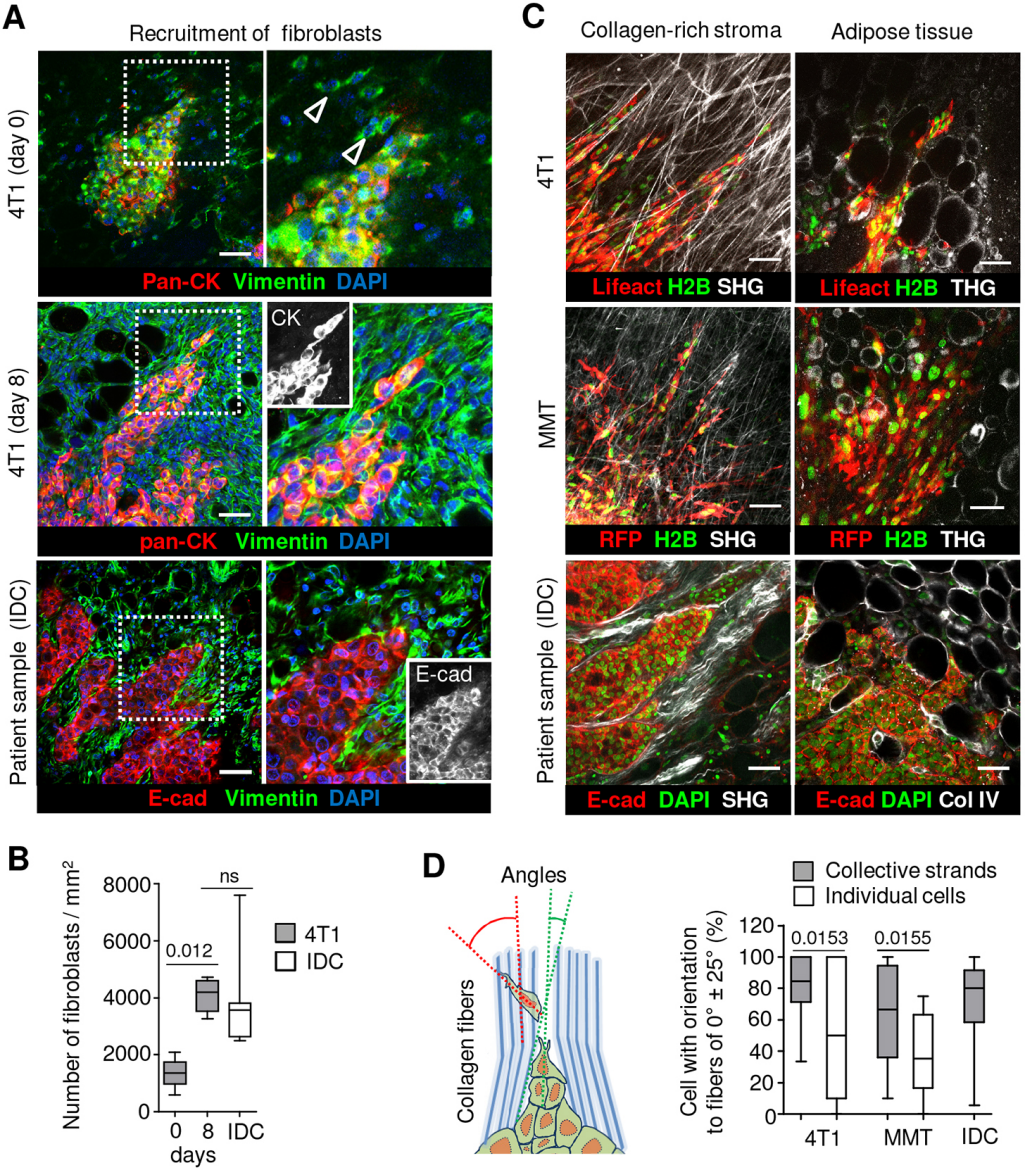
**Stroma remodeling in implanted mammary tumors and patient samples**. (A) Example images representing the stromal composition in the murine mammary fat pad directly after 4T1 spheroid implantation (upper row), and after 8 days (middle row), compared with human invasive ductal carcinoma (IDC) lesion (lower row). Arrowheads indicate vimentin-positive fibroblasts. (B) Number of vimentin-positive stromal cells in the peritumoral stroma. Data per group represent 5 tumors from 3 mice and 4 independent human IDC samples. Box plots in B display the median (black line), 25/75 percentiles (boxes) and maximum/minimum (whiskers). *P*-values were obtained by the Mann–Whitney test. (C) Collective and single-cell invasion of 4T1 (upper row) and MMT (middle row) cells 4 days after spheroid implantation, compared with human IDC (lower row) invading collagen-rich stroma and adipose tissue. (D) Principle of analysis (left) and quantification of tumor cell alignment along collagen fibers (right). Cells and collagen fibers in 4T1 and MMT tumors 4 days after spheroid implantation and human IDC were identified as shown in C. Quantification of cell alignment relative to collagen fibers, expressed as a fraction within the 0±25° sector. Data represent 34 (4T1) and 21 (MMT) collective strands, and 21 (4T1) and 17 (MMT) single cells, from 8 tumors from 3 mice per cell line, and 35 collective strands from 5 independent human IDC samples. Box plots in B and D display the median (black line), 25/75 percentiles (boxes) and maximum/minimum (whiskers). *P*-values were obtained by the Mann–Whitney test. Scale bars: 50 μm.

In the window model, tumor growth and invasion were accompanied by neo-angiogenesis (Fig. 2A,D) and notable accumulation of fibroblasts at the tumor-stroma interface, similar to human samples (Fig. 3A,B). We mapped the 3D tissue topology next to, and ahead of, the invasion margin to address whether early-onset collective invasion follows microenvironmental structures, a process identified in individually moving breast cancer cells in genetically engineered breast cancer and collectively invading mesenchymal tumors (Gligorijevic et al., 2014; Weigelin et al., 2012). Collective strands, including tip cells, were often aligned parallel to collagen bundles, recapitulating alignment of multicellular strands along stromal collagen in human lesions (Fig. 3C). However, whether early-onset collective invasion causes remodeling or rather follows pre-existing aligned collagen fibrils *in vivo* is not known. By comparison, individually located 4T1 and MMT cells showed more variable, loosened angle distribution and alignment along collagen structures (Fig. 3D; Fig. S2F). These data suggest that collective invasion follows more precisely aligned collagen and interfaces, whereas detached single cells are more likely to change direction between guiding tissue structures. Thus, implanted microlesions reproduce collective and individual carcinoma invasion modes and their guidance by stromal structures (Schedin and Keely, 2011).

### Adaptive actin dynamics during collective to single-cell transition

**Fig. 4.**
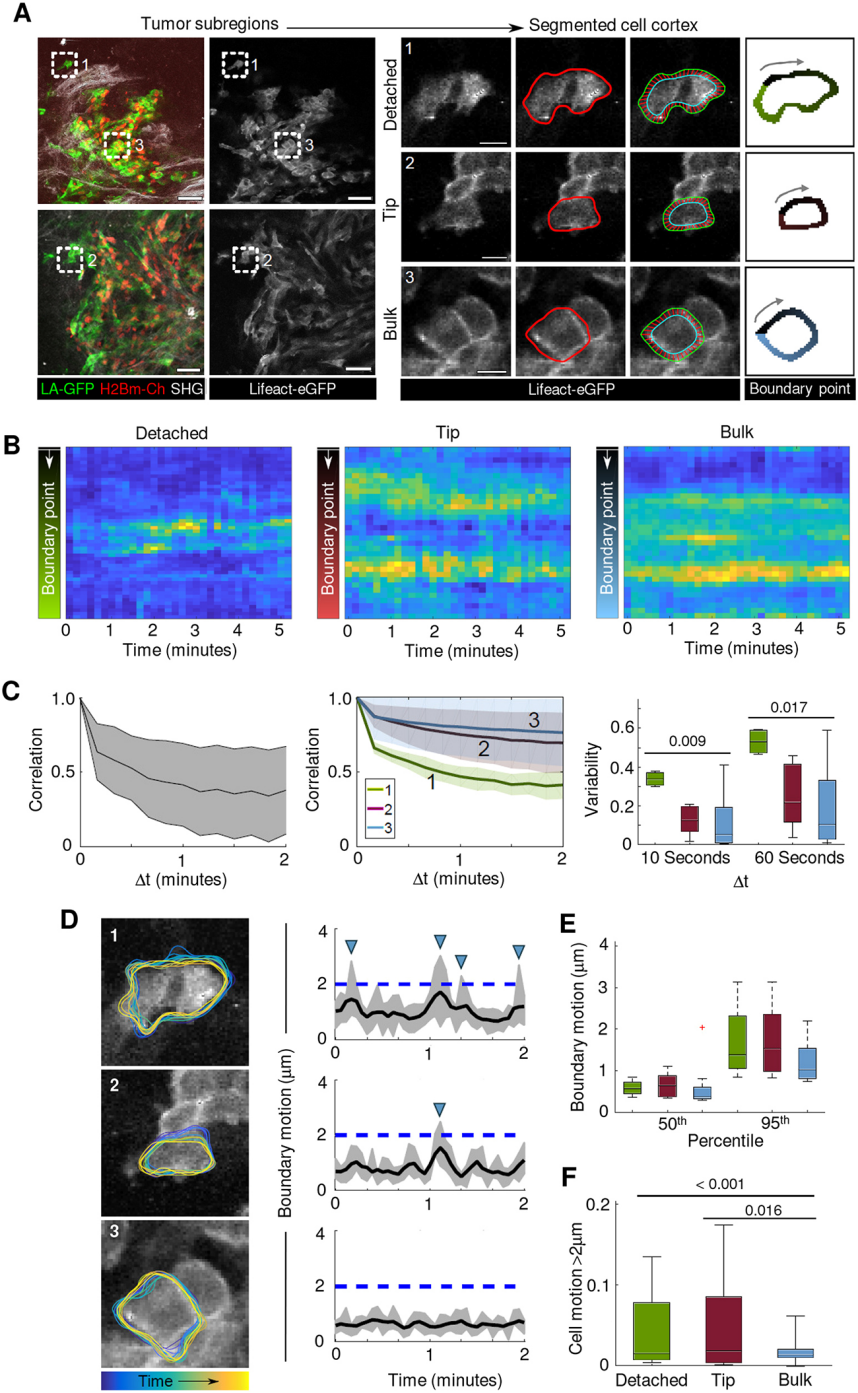
**Actin dynamics during collective and single-cell invasion.** The time-dependent shape of each cell was derived from sequential single imaging planes, extracted by semi-automatic shape analysis, and the Lifeact-eGFP intensity along the cell periphery was obtained from equal segments 2.07 µm inward from the boundary. (A) 4T1 cells at different invasion position *in vivo* 4 days after implantation. Segmented cells were categorized as detached (1), tip (2) and bulk cells (3), and a topology of boundary points was calculated, labeled and tracked for time-dependent analysis of actin intensity. (B) Kymograph of mean actin intensity in each boundary point section over time. (C) Actin fluctuations in invading cells. Left: time-delay autocorrelations of actin intensity derived from the example detached cell shown in B (the black line is the mean and the gray region represents standard deviation). Middle: aggregated time-delay autocorrelation curves of actin intensity for detached (1), tip (2) and bulk (3) cells, represented as the mean (black lines) and standard deviation (shaded areas) from 4, 8 and 15 cells, respectively, from 3 independent tumors. Right: intensity variability for intervals of 10 s (representing fast fluctuations) and 60 s (slow fluctuations) within detached, tip and bulk cells. Significant differences were found when comparing the mean variability between detached and bulk cells. (D) Cell boundary motion traces and their boundary motion distributions obtained from time-lapse sequences for detached (1), tip (2) and bulk (3) cells. Each group of cells exhibits similar background behavior despite differences in actin intensity variability, with detached and tip cells experiencing a higher frequency of large protrusion events than bulk cells. This trend is further indicated in the box plots (E) of the distribution of the 50th and 95th percentiles of boundary motion, albeit no significant differences amongst means were found. (F) The percentages of motion above 2 µm for the indicated cell subsets indicate a significant difference in behavior between detached and bulk, and tip and bulk, cells. Box plots in C, E and F show the median (black line), 25/75 percentiles (boxes), maximum/minimum (whiskers) and outlier (red cross) from 5-6 cells per condition from 3 independent experiments. *P*-values were obtained by Bonferroni corrected one-way ANOVA and the Tukey–Kramer multiple comparison test. Scale bars: 50 μm (A, overviews), 10 μm (A, areas from dashed line boxes shown magnified on the right).

Plasticity of cancer cell invasion is associated with an adaptive actin cytoskeleton. *In vitro*, cortical actin networks define the shape, polarity and force transmission in individually moving cells (Hidalgo-Carcedo et al., 2011), and further stabilize adherens junctions and transmit long-range forces across connected cells during collective migration (Gomez et al., 2011). To address whether actin dynamics in individual and collective invasion differ *in vivo*, the intensity fluctuations of Lifeact-eGFP in 4T1 tumors transiting from collective to single-cell migration were quantified (Fig. 4A,B). Local actin filament stability, measured by time-delay autocorrelations of Lifeact-eGFP intensity, was high in cells inside (bulk cells) and at the edge (tip cells) of collective invasion zones, but significantly decreased in detached cells (Fig. 4C). This indicates an upregulation of actin dynamics after individualization from the collective invasion zone.

*In vitro*, amoeboid cells moving along extracellular matrix substrate develop actin waves reaching speeds of ∼10 µm/min (Driscoll et al., 2014), with calculated changes in polymerization on timescales of seconds (Giannone et al., 2007), whereas cell protrusions, such as ruffles or pseudopods, remodel at slower timescales (minutes) (Giannone et al., 2007). To test whether actin dynamics *in vivo* reach similar kinetics, we probed the intensity variations for 10 and 60 s time delays in collective and single-cell migration *in vivo*. Detached cells showed a sharp decay of autocorrelation compared with bulk or tip cells (Fig. 4D,E), indicating increased variability of actin dynamics. Likewise, cortical Lifeact-eGFP was focally enriched in individual cells (Fig. 4A) and associated with larger protrusions (>2 µm) and more frequent protrusion growth than in bulk cells, whereas tip cells developed an intermediate protrusion size and frequency (Fig. 4F). In aggregate, these *in vivo* data suggest an association between increased actin dynamics and decreased precision of tissue guidance after collective to single-cell transition.

## DISCUSSION

Although collective invasion patterns are abundantly detected in clinical samples of epithelial cancers, including breast carcinoma (Bronsert et al., 2014; Cheung et al., 2013; Khalil et al., 2017), current intravital microscopy models of breast cancer were insufficient to reliably detect and mechanistically interrogate collective invasion (Giampieri et al., 2009; Gligorijevic et al., 2014; Kedrin et al., 2008). Reasons for this model insufficiency are likely multifactorial, based on (1) lack of cell-cell junctions when tumor cell suspensions are implanted; (2) excessive growth, which might cause circular tissue compression and disable multicellular invasion; and (3) a bias in genetic mouse models, which disable or minimize cadherin-based cell-cell junctions and possibly favor individual cell, but not collective, behaviors. To overcome this problem, we used orthotopic microimplantation of multicellular spheroids of murine breast cancer cells into the mammary fat pad/stroma interface, and monitored – by intravital microscopy – reliable collective invasion followed by transition to individual cell detachment *in vivo*.

Using spheroid microimplantation at the edge of the mammary fat pad, this intravital microscopy approach captures critical early steps of collective invasion and cytoskeletal plasticity in breast cancer tumors. With transition from collective to single-cell migration, individually disseminating cells gain oscillatory behavior of the actin cytoskeleton and abandon strict alignment along collagen fibers. Such collective to single-cell transitions arguably broaden a tumor cell's ability to cope with different tissue topologies and escape from the primary site (Friedl and Alexander, 2011).

In both E-cadherin-positive and E-cadherin-negative cells, implanted spheroids support tumor-like topology and cell-cell cohesion for emerging collective invasion followed by gradual individualization. E-cadherin is considered an important gatekeeper of epithelial functions. Because single-cell events are enhanced in epithelial tumors when E-cadherin is downregulated (Khalil et al., 2017), E-cadherin was initially considered to counteract local tissue invasion and metastatic spread (De Craene and Berx, 2013). However, in clinical breast cancer, the E-cadherin status does not predict metastatic outcome and prognosis (Khalil et al., 2017; Narendra et al., 2015). Similar to these clinical data, E-cadherin-negative MMT cell tumors, albeit developing more frequent cell individualization compared with E-cadherin-expressing 4T1 tumors, did not give rise to increased rates of distant metastasis. Beyond mechanical cell-cell cohesion, other pathways might control the metastatic outcome in MMT and 4T1 cells, including oncogenic signaling, sensitivity to growth factors and mechanoregulation (Friedl and Alexander, 2011; Giampieri et al., 2009; Harney et al., 2015).

Our data further reveal plasticity of the actin cytoskeleton during collective to single-cell transition *in vivo*, as predicted from *in vitro* models and *in silico* (Hidalgo-Carcedo et al., 2011; Te Boekhorst et al., 2016). A relatively quiescent actin cytoskeleton in the collective invasion zone possibly fulfills a dual function in (1) controlling both the stability and turnover of cell-cell junctions, and (2) providing a starting point for actin-rich protrusions at the invasion front. Leader cells which extended outwards to the stroma still retained overall quiescent actin dynamics, comparable to those of bulk cells, but developed large protrusions at rates comparable to those in individual cells. Likely as a consequence of detachment, actin dynamics in individually moving cells unleash oscillations, indicating that the actin cytoskeleton in invading tumor cells is an excitable system with dynamic focalization and protrusion bursts, not unlike traveling actin waves in moving *Dictyostelium discoideum* amoebae and fibroblasts migrating on 2D substrate *in vitro* (Driscoll et al., 2012; Giannone et al., 2007). Combined with fate tracing of distinct invasion modes, we consider spheroid microimplantation as a potentially suitable model to barcode invasion mechanisms and stromal niches in primary lesions, and derive their relevance for distant metastasis formation and resistance to therapy (Brighton et al., 2018; Hirata et al., 2015).

When benchmarking *in vitro* and *in vivo* analyses of cancer cell invasion, the spheroid microimplantation strategy closes a ‘validation gap’, as it reflects invasion types and plasticity derived from both 3D organotypic culture and clinical samples (Bronsert et al., 2014; Khalil et al., 2017). Multiple spheroids can be implanted in the same mouse to increase the surface area of the tumor-stroma interface and collect larger data sets. Multiple microlesions further increase the tumor mass and enable spontaneous distant metastasis from these lesions, and this reduces animal consumption and improves data quality from the same mouse. Because intact spheroids are implanted, the strategy will be amenable for epithelial cancer organoids or dissected tumor microtissues from patients to create mouse avatars for personalized medicine.

## MATERIALS AND METHODS

### Antibodies and reagents

Primary antibodies and dilutions were as follows: anti-human E-cadherin (13-5700, mouse monoclonal, clone SHE78-7, Thermo Fisher Scientific; 1:100); anti-human E-cadherin (610181, mouse monoclonal, clone 36/E, BD Biosciences; 1:100); anti-human E-cadherin (MA5-14458, rabbit monoclonal, clone EP700Y, Thermo Fisher Scientific; 1:100); anti-mouse N-cadherin (C3865, mouse, clone GC-4, Sigma-Aldrich; 1:200); anti-human beta-catenin (610153, mouse monoclonal, clone 14/beta-catenin, BD Biosciences; 1:200); anti-mouse p120 catenin (610133, mouse monoclonal, clone 98/pp120, BD Biosciences; 1:200); anti-human cytokeratin 8 (ab53280, rabbit monoclonal, clone EP1628Y, Abcam; 1:2000); anti-bovine wide spectrum cytokeratin (ab94894, rabbit polyclonal, Abcam; 1:100); anti-rat vimentin (ab24525, chicken polyclonal, Abcam; 1:300); anti-human collagen IV (PAI-28534, rabbit polyclonal, Thermo Fisher Scientific; 1:200). Secondary antibodies were Alexa Fluor 488/546/647-conjugated goat anti-mouse, anti-chicken or anti-rabbit (cross-adsorbed) (Thermo Fisher Scientific; 1:200). F-actin was visualized with Alexa Fluor 568-conjugated phalloidin (Thermo Fisher Scientific; 1:200) and nuclei were labeled with 4′,6-diamidino-2-phenylindole-(DAPI) (1 µg/ml, Roche Diagnostics). Blood vessels were visualized by intravenous injection of Alexa Fluor 750-labeled 70 kDa-dextran (2 mg/mouse, Thermo Fisher Scientific).

### Cell lines and culture

Mouse mammary carcinoma cells 4T1 [CRL-2539, American Type Culture Collection (ATCC)] and MMT (MMT 060562, ATCC) expressing cytosolic DsRed2 and nuclear H2B-eGFP (kindly provided by Dr R. M. Hoffman, AntiCancer, San Diego, USA) were maintained in RPMI 1640 growth medium (Thermo Fisher Scientific) supplemented with 10% fetal bovine serum (Sigma-Aldrich), 1% sodium pyruvate (Thermo Fisher Scientific), 100 U/ml penicillin and 100 μg/ml streptomycin (PAA Laboratories), and 2 mM L-glutamine (Thermo Fisher Scientific) (37°C, 5% CO_2_, humidified atmosphere). The identity of tumor cells was verified by short tandem repeat (STR) DNA profiling (IDEXX BioResearch). No mammalian interspecies contamination was detected. The STR DNA profile of MMT cells was generated, for the first time, as follows: MCA-4-2, 21.3 NA; MCA-5-5, 14 NA; MCA-6-4, 18 NA; MCA-6-7, 13 NA; MCA-9-2, 15 NA; MCA-12-1, 16 NA; MCA-15-3, 22.3 NA; MCA-18-3, 19 NA; MCA-X-1, 24 NA. This profile does not match any previously reported profile. Cells were routinely tested for mycoplasma contamination (Myco Alert, Lonza). Lentiviral particles were produced in HEK293T cells by the ViraPower expression system (Life Technologies), according to the manufacturer's protocol. H2B-mCherry was subcloned from pcDNA 3.0 H2B-mCherry (a gift from Dr A. J. C. de Groof, Radboud University Medical Center, Nijmegen, The Netherlands) into the lentiviral backbone pLenti6.2v5 (Life Technologies). To generate dual-color cells, pLentiCMV-MCS-Lifeact-eGFP vector (a gift from Dr Olivier Destaing, Institute Albert Bonniot, Grenoble, France) was introduced into H2B-mCherry-expressing 4T1 cells. Stable clones were established by selection with blasticidin (5 μg/ml) and puromycin (3 μg/ml) followed by single-cell sorting (FACS Aria SORP, Becton Dickinson).

### Spheroid generation

Spheroids were generated using the hanging-drop method. Cells from subconﬂuent cultures were detached with 1 mM EDTA and 0.075% trypsin (Thermo Fisher Scientific), counted, and 500-1000 cells were incubated in 25 µl droplets of RPMI 1640 growth medium supplemented with 0.12% methylcellulose (Sigma-Aldrich) (18 h, 37°C, 5% CO_2_, humidified atmosphere). After cell aggregation, spheroids were harvested, washed and maintained in Dulbecco's phosphate buffered saline (PBS) containing 0.9 mM Ca^2+^ and 0.5 mM Mg^2+^ (Thermo Fisher Scientific) for subsequent analysis.

### Spheroid invasion into 3D collagen lattices

Spheroids were incorporated into type I collagen from rat-tail tendon (BD Biosciences; ﬁnal concentration 4 mg/ml) prior to polymerization at pH 7.4 according to the manufacturer's protocol and overlaid with complete growth medium prior to invasion culture (48 h).

### Primary breast cancer samples

Tissue samples from 5 invasive ductal carcinoma (IDC) and 3 invasive lobular carcinoma (ILC) patients were obtained after surgical removal of primary breast cancer (Department of Pathology, Radboudumc, Nijmegen, The Netherlands). Tumor samples were encrypted and analyzed in an anonymized manner, as approved by the Institutional Review Board and according to national law and in compliance with the Declaration of Helsinki (Nagelkerke et al., 2011). According to Dutch legislation, no approval from a research ethics committee was required for this study, as coded tissue obtained from routine diagnostic workflow was used and the included patients were not affected by the study. Anonymous or coded use of redundant tissue for research purposes is part of the standard treatment agreement with patients in Radboudumc Hospital, from which patients may opt out. None of the included patients submitted an objection against use of residual material. Patient material was used in a manner compliant with the Declaration of Helsinki.

### Immunohistochemistry

Paraffin-embedded tissue sections were deparaffinized before antigen retrieval in Tris-EDTA buffer (pH 9.0, 95°C, 15 min), and then incubated with 3% hydrogen peroxidase [10 min, room temperature (RT)], washed in 0.1 M Tris/HCl buffer (pH 7.5, 3×, 5 min), incubated with primary antibody (4°C, overnight), washed (3×, 10 min), incubated with a secondary antibody conjugated to a polymer labeled with horseradish peroxidase (30 min, RT), washed (3×, 10 min) and incubated with diaminobenzidine (DAKO; 5 min, RT), followed by Hematoxylin staining (1-2 min). Sections were mounted in Pertex medium (Histolab) and scanned (Pannoramic 250 Flash II, 3DHistech).

### Confocal microscopy

For immunofluorescence staining, collagen-embedded spheroids were fixed in 4% paraformaldehyde, incubated with primary antibody dissolved in PBS/0.1% bovine serum albumin/0.1% Triton X-100 (Sigma-Aldrich) (12 h, 4°C), washed (PBS, 3×, 30 min), incubated with secondary antibody, phalloidin and DAPI (2 h, RT), and embedded in a customized imaging chamber.

Thick tissue slices (200 μm) were obtained by vibratome sectioning (Leica, VT100s) of formalin-fixed breast cancer samples or mouse mammary fat pads. After antigen retrieval in Tris-EDTA buffer (pH 9; 95°C, 15 min), tissue slices were incubated with primary antibody, washed 5× and incubated with secondary antibodies and DAPI. All washing and incubation steps were performed in PBS containing 0.1% Triton-X 100, 2% goat serum and 0.05% sodium azide (24 h, 20°C).

Confocal microscopy (Olympus FV1000) was performed using 20×/0.50 NA and 40×/0.80 NA long working distance objectives. Two-photon microscopy (LaVision BioTec) of fixed 3D tissue samples was performed using a 20× Olympus XLUMPlanFI 206/0.95 objective.

### *In vivo* spheroid implantation and mammary window imaging

Balb/C (for 4T1 tumors) and BALB/C-nu/nu (CAnN.Cg-Foxn1nu/Crl; for MMT tumors) female mice (6-8 weeks old) were obtained from Charles River Laboratories. Animal procedures were approved by the Animal Ethics Committee of Radboud University, Nijmegen (RU-DEC 2012-129) and performed according to the guidelines of the Dutch Act on Animal Experimentation and the European FELASA protocol.

For implantation of the mammary window, the 4th mammary fat pad in anesthetized mice (1-2% isoflurane in oxygen) was exposed by surgical incision using microsurgery under a Leica MZFLIII microscope. A superficial microchannel was created within the fat pad using a 30 G syringe needle (BD Medical) into which an individual spheroid in a microdrop (2 µl in PBS) was implanted using a gel-loading pipet tip (0.3 mm inner diameter, BIOplastics). After implantation of up to 10 spheroids in the same tissue area, a mammary imaging window was inserted, affixed to the skin by gentle tightening of a purse-string suture around the skin rim, and closed with a cover glass and spring ring, as described (Alieva et al., 2014). Multifocal tumor development was monitored by whole-body fluorescence imaging (FluorVivo100, INDEC BioSystems) and quantified as total tumor area in the mammary window from 2D projections of the DsRed2 and H2B-eGFP signal.

### Intravital 2-photon microscopy

Intravital imaging was performed on a customized upright TrimScope II multiphoton microscope (LaVision BioTec) equipped with 3 tunable Ti:Sa lasers and an Optical Parametric Oscillator (Coherent/APE) using a 20× Olympus XLUMPlanFI 206/0.95 water-immersion objective and up to 5 detectors with differential filter configuration (395/8 or 447/60, blue; 525/50, green; 593/11, 593/40 or 620/60, red; 675/67, 710/75 or 810/90, far red; Semrock or Chroma Technology). The mammary imaging window of anesthetized mice (1-2% isoflurane in oxygen) was stabilized by a custom holder on the temperature-controlled stage (37°C). Blood vessels were visualized by intravenous injection of 70 kDa dextran conjugated to Alexa Fluor 750 (2 mg/mouse, Molecular Probes). Multichannel recordings were acquired as 3D stacks and time-lapse movies by sequential excitation at 910 nm (eGFP), 1090 nm (second harmonic generation, DsRed2, mCherry, Alexa Fluor 750) and 1180 nm (third harmonic generation) with 20-60 mW average power under the objective for up to 4 h observation periods.

### Spontaneous metastasis assay

4T1 or MMT tumors were grown by implanting 10 spheroids (1000 cells each) into the same mammary fat pad within the mammary imaging window. Two to three weeks after spheroid implantation, the mice were sacrificed and primary tumors and lungs were harvested, snap-frozen in liquid nitrogen (MMT) or fixed in buffered formalin (4%, 24 h) followed by embedding in paraffin (4T1). The absolute number of metastatic events of dual-color MMT cells was counted by sectioning whole lungs into 14 µm slices, with intervals of 70 µm, followed by staining with DAPI and whole-field fluorescence scanning (Pannoramic 250 Flash II) of DAPI, eGFP and DsRed2. For mice bearing 4T1 tumors, which showed less reliable fluorescent protein expression after 3 weeks of growth *in vivo*, tumor cells were identified by staining for cytokeratin 8 and counted by sectioning whole lungs into 5 µm slices, with intervals of 70 µm, followed by whole-slice scanning (Pannoramic 250 Flash II). Normal lung cells (i.e. pneumocytes) were also detected by cytokeratin 8 staining, and were excluded from quantification based on nonclustered cell morphology, position in alveoli, and smaller size and regular shape of the nucleus compared with nuclei of 4T1 cancer cells. Metastatic load was calculated as the average number of metastatic cell clusters per tissue slice from 74-85 (4T1) and 85-124 (MMT) slices per lung. As a minimum size for inclusion in the analysis, ≥3 4T1 and MMT cells were considered as a metastatic cluster.

### Image processing and analysis

Images were obtained using a *z*-step size of 5 µm, represented as a single slice, maximal projection and 3D reconstruction using Fiji (1.49s, National Institutes of Health). 3D stacks were stitched (mosaic plugin), drift corrected during time-lapse recording (StackReg plugin) and manually segmented to calculate the tumor area. The area of blood vessels was obtained by automated single-channel thresholding of the dextran channel (Alexa Fluor 750) representing perfused blood vessels and normalized to the tumor area. Collagen fiber alignment relative to invading collective strands or individual cells was quantified as the angle between both respective length axes.

### Analysis of actin dynamics

Actin dynamics were measured as fluctuations of Lifeact-eGFP fluorescence intensity in space and time from single imaging planes through the center of the cell body without signs of drift. Using a custom semiautomatic MATLAB code (MathWorks), the cell bodies were segmented, and the boundary motion and variability of cortical actin was calculated based on source images with 3.02 µm or 1.45 µm pixel resolution and adjusted signal-to-noise ratio, as described (Driscoll et al., 2014). The cell boundary was defined with subpixel accuracy using an automatic snake algorithm with 200 boundary points. The edge layer of the cell body was defined by subtracting an eroded binary mask of the cell body from a full mask of the cell body. Boundary motion over time was detected by performing a least squares fitting to match boundary points of subsequent frames. The variation of cortical Lifeact-eGFP was calculated for regions near each boundary point along the cell boundary. The boundary length per region was 1.73 µm, which was larger than the average fluctuations in measured boundary position and thus sufficient to capture cortical actin with accuracy. The mean intensity per boundary region over time was plotted as a kymograph and used for calculating the time-delay autocorrelation, defined as:
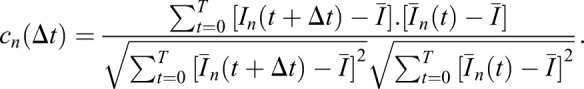


In this equation, *n* represents the boundary index and 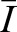 an intensity offset to subtract the mean background fluorescence within each cell. The resulting time-delay autocorrelation functions were averaged for given time delays (Δ*t*). Intensity variability was calculated as 1 minus the time-delay autocorrelation represented by 1-*c*_*n*_(Δ*t*). Because a correlation of 1 indicates complete stability of cortical actin with time, this parameter corresponds to the time variability of cortical actin.

### Statistics

Statistical analysis was performed with GraphPad software v.5, using the nonparametric Mann–Whitney test, Bonferroni-corrected one-way ANOVA and the Tukey–Kramer test for multiple comparisons. *P*-values less than 0.05 were considered significant.

## Supplementary Material

Supplementary information
