# Supplementary material for: Intravital microscopy of collective invasion plasticity in breast cancer
Source: Dis Model Mech. 2018 Aug 23;11(9):dmm034330. doi: 10.1242/dmm.034330 (PMC6176993; doi:10.1242/dmm.034330)
Supplement: Supplementary information [file dmm-11-034330-s1.pdf]

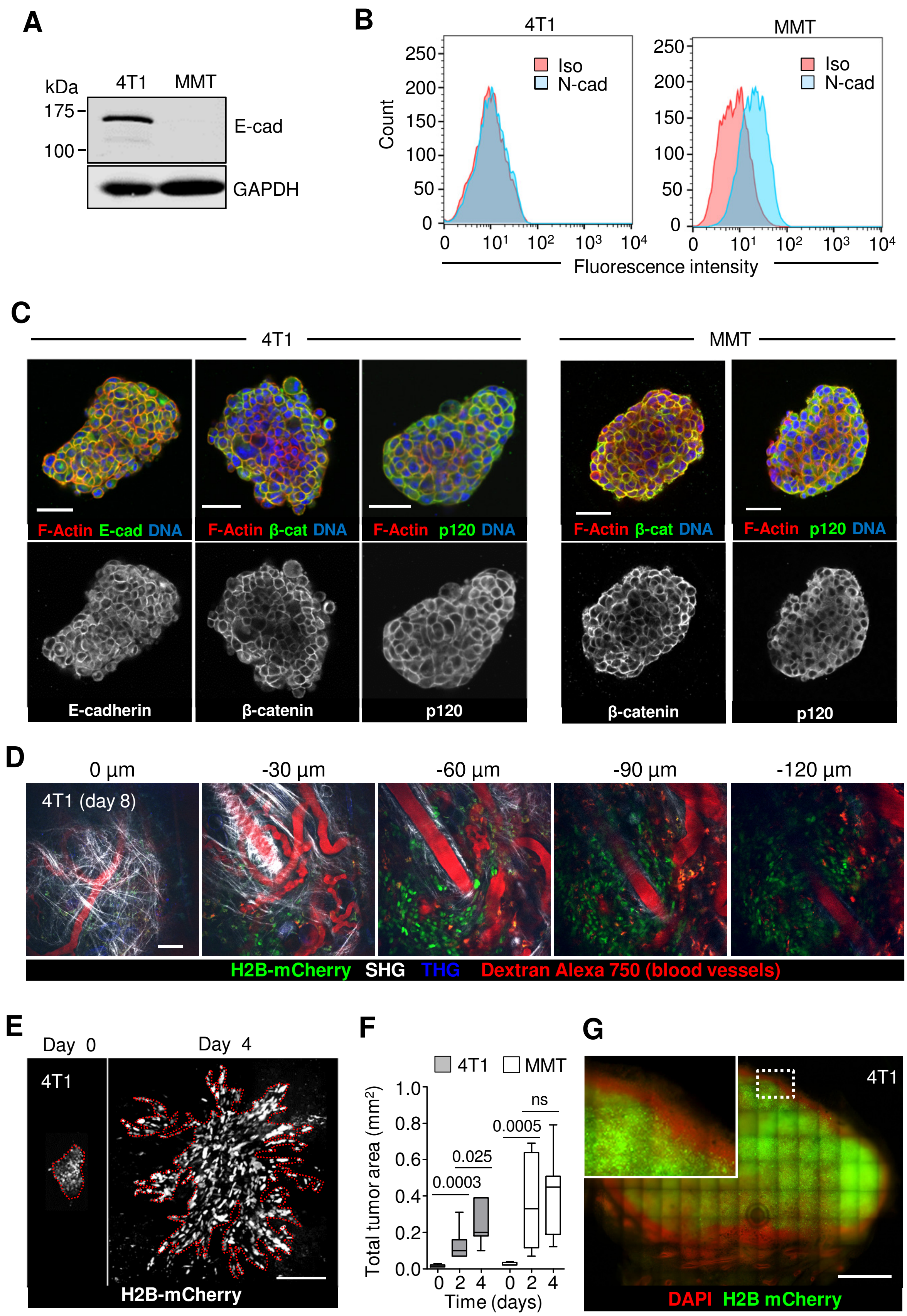

Disease Models & Mechanisms • Supplementary information

Supplementary figure 1. 4T1 and MMT spheroid models and kinetics *in vivo*. **A**, E-cadherin protein expression detected by western blot in 4T1 and MMT cells cultured in vitro. **B**, Expression of N-cadherin in 4T1 and MMT cells measured by flow cytometry. **C**, E-cadherin, β-catenin and p120 catenin distribution along cell-cell junctions of spheroids prior to their implantation *in vivo*. **D**, Individual slices at different z-depths of the tumor within the mammary fat pad 8 days after spheroid implantation. **E**, Increase of 4T1 tumor area (H2B-mCherry labelling tumor nuclei) between 0 to 4 days after spheroid implantation. The red line represents the segmented region for area analysis in (F). **F**, Quantification of 4T1 and MMT tumor areas over time. Data represent the median (black line), 25/75 percentiles (boxes) and maximum/minimum (whiskers) of 3-5 tumors from 3 mice per cell line. P-values were obtained by the Mann-Whitney test. **G**, 4T1 tumor in the mammary fat pad 8 days after implantation of 10^5 cells in suspension. Scale bars, 50 μm (C, D); 200 μm (E); 1000 μm (G).

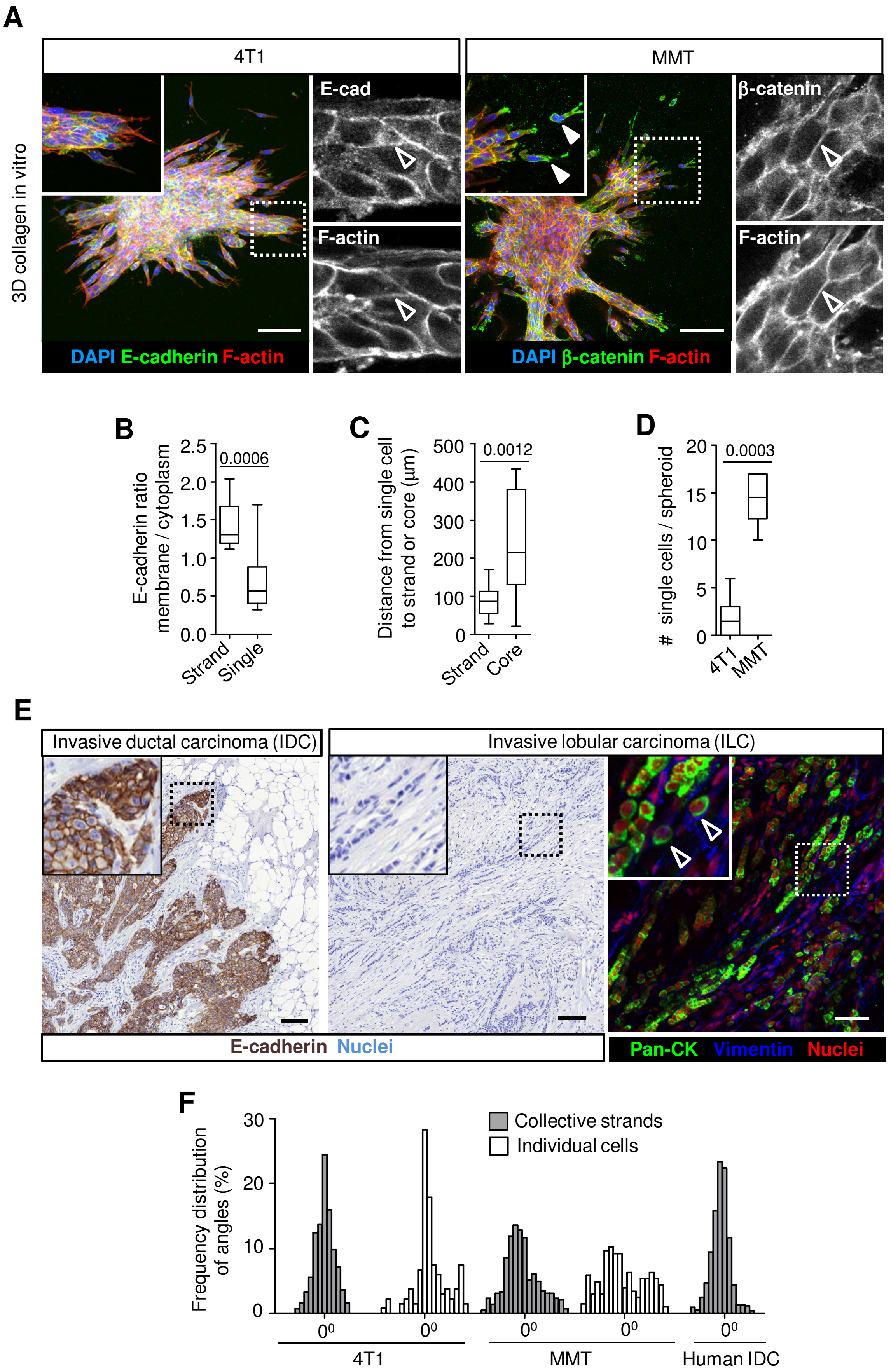

Disease Models & Mechanisms • Supplementary information

Supplementary figure 2. Collective invasion of E-cadherin positive and negative breast cancer cells in 3D spheroid culture, mammary imaging model and clinical samples. **A**, Collective and single cell invasion from 4T1 and MMT spheroids in 3D collagen matrix in vitro 48 h after spheroid embedding. Junction proteins E-cadherin and β-catenin are present along cell-cell junctions (arrowheads) in collective strands. Zoomed insets, detaching single cells (white arrowheads). **B**, Membrane vs cytoplasmic E-cadherin levels in 4T1 collective strands and single cells invading from spheroids in 3D collagen in vitro. Data represent 19 (strand) and 9 (single) cells from 3 independent experiments. **C**, The distances from single 4T1 cells to the nearest tip cell in collective strand or to the spheroid core in mammary imaging model 4 days after spheroid implantation. Data represent 26 single cells from 6 tumors in 3 mice. **D**, The number of individually invading 4T1 and MMT cells per spheroid in 3D collagen in vitro. Data represent 18 (4T1) and 6 (MMT) spheroids from 2 independent experiments. Values in (B-D) display the median (black line), 25/75 percentiles (boxes) and maximum/minimum (whiskers). P-values were obtained by the Mann-Whitney test. **E**, Representative clinical primary IDC and ILC samples with stroma invasion by multicellular strands/files with or without E-cadherin expression, respectively. Immmunohistochemistry of E-cadherin expression in IDC and ILC lesions (left and middle panels). 3D reconstruction of 200 µm-thick tissue (ILC; right panel) showing collective files and individual cells (arrowheads). Carcinoma cells are visualized with pan-cytokeratin (pan-CK) and stromal cells with vimentin staining. **F**. Frequency distribution of angles between fibrillar collagen and collective strands or individual cells in 4T1 and MMT tumors within the mouse mammary fat pad, and in human IDC lesions (related to Fig. 3D). Data represent 34 (4T1), 21 (MMT) collective strands and 21 (4T1), 17 (MMT) single cells of 8 tumors from 3 mice per cell line, and 35 collective strands from 5 independent human IDC samples. The angles were measured as described in Fig. 3D. Scale bars, 100 µm.
